# Do the Th17 Cells Play a Role in the Pathogenesis of Leptospirosis?

**DOI:** 10.1155/2018/9704532

**Published:** 2018-07-10

**Authors:** Kanchana Bandara, Chinthika Gunasekara, Manjula Weerasekera, Chamil Marasinghe, Nilantha Ranasinghe, Neluka Fernando

**Affiliations:** ^1^Department of Basic Sciences, Faculty of Allied Health Sciences, General Sir John Kotelawala Defence University, Ratmalana, Sri Lanka; ^2^Department of Microbiology, Faculty of Medical Sciences, University of Sri Jayewardenepura, Gangodawila, Nugegoda, Sri Lanka; ^3^Department of Medicine, Faculty of Medical Sciences, University of Sri Jayewardenepura, Gangodawila, Nugegoda, Sri Lanka; ^4^Out Patients Department, Base Hospital, Horana, Sri Lanka

## Abstract

**Objectives:**

The aim of this study was to determine the level of five different pro- and anti-inflammatory cytokines to study the inflammatory response of leptospirosis.

**Materials and methods:**

The serum cytokine levels of IL-10, IL-17A, IL-21, IL-23, and TNF-*α* were investigated in 57 patients with leptospirosis and 12 healthy controls using a commercially available ELISA kit (Mabtech, Sweden). Statistical analysis was done using Graphpad Prism.

**Results:**

Elevation of serum IL-10 and IL-17A levels and significant elevation of serum IL-21 (*p*=0.002), IL-23 (*p*=0.002), and TNF-*α* (*p*=0.039) were observed among leptospirosis patients compared to the healthy control group. The two major complications observed among these patients were renal failure and liver involvement. Renal failure was significantly associated with elevation of IL-21 and IL-23, while patients with liver involvement had a significant elevation of IL-21, IL-23, and TNF-*α*.

**Conclusion:**

Elevation of IL-17A together with the significant elevation of IL-21 and IL-23 suggests a possible involvement of Th17 cells in the immunopathogenesis of leptospirosis.

## 1. Introduction

The clinical presentation of leptospirosis varies from mild flu-like illness to severe multiorgan failure and death. The reasons for severe clinical form are still an enigma and possibly multifactorial. The host immune response plays a definitive role in determination of the disease severity as leptospirosis is associated with a strong inflammatory reaction. Structural components of *Leptospira* have been shown to induce proinflammatory cytokines [1]. Several proinflammatory and anti-inflammatory cytokines, colony-stimulating factors, and chemotactic factors are expressed during acute infection [2]. Cytokines released by infected or activated cells are important mediators of host immunity leading to protective immunity or pathology. Therefore, cytokine response could be a useful marker in understanding the pathogenesis in leptospirosis and thereby the outcome of the disease.

Although several studies have been conducted to evaluate the cytokine response with regard to TH1/TH2 cell responses, very few have investigated the TH17 arm of T-cell response in patient populations [3]. Recently, TH17 cells have emerged as playing an important role in immunity to extracellular pathogens including bacteria and fungi. As leptospirosis is associated with strong inflammatory reaction, it is strongly possible that TH17 cells play a role in disease pathogenesis. TH17 cells secrete several proinflammatory cytokines including IL-17 and TNF-*α* which are involved in the recruitment of neutrophils to the site of infection contributing to tissue destruction.

TH17 cell differentiation occurs in the presence of IL-21. The maintenance of the differentiated TH17 cells is carried out by cytokine IL-23. A main proinflammatory cytokine secreted by the TH17 cells is IL-17 which acts on various signaling pathways to upregulate the expression of proteins. Furthermore, TNF-*α* is also secreted by the TH17 cells. Only few studies have investigated the role of IL-17 among leptospirosis patients [2]. Different cytokine patterns have been reported among leptospirosis patients presenting with mild symptoms and in those developing complications [4]. Therefore, it is important to determine the expression of these cytokines in order to support the hypothesis that TH17 cells play a role in the pathogenesis of leptospirosis.

## 2. Materials and Methods

### 2.1. Study Design

A descriptive cross-sectional study was conducted at the medical wards of selected hospitals in Western and Southern provinces of Sri Lanka between January 2013 and January 2015. Ethical approval for the study was obtained from the Ethical Review Committee of University of Sri Jayewardenepura (no. 702/12).

### 2.2. Study Sample

Fifty-seven adult inward patients who had confirmed diagnosis of leptospirosis by a MAT titre ≥1 : 400 or PCR [5] were recruited for the study following the WHO guidelines [6]. Patients with diagnoses other than leptospirosis and patients less than 18 years of age were excluded from the study. Twelve healthy controls were recruited by an open advertisement. Blood was collected from the patients suspected of leptospirosis, and serum was separated and stored at −80°C.

### 2.3. Data Collection

Clinical data of patients were gathered from the BHT with the aid of a pretested questionnaire. Sociodemographic profile, clinical features, risk factors, basic laboratory findings (FBC, urine full report, serum electrolytes, renal profile, liver profile, ECG, and ECHO), and outcome/complications of the patient were recorded. Patients with confirmed diagnosis of leptospirosis were categorized into the following groups: patients with/without renal failure and patients with/without liver involvement.

### 2.4. Enzyme-Linked Immunosorbent Assay (ELISA) for Cytokines

Five ELISA assays were used to quantitate the levels of cytokines IL-10, IL-17A, IL-21, IL-23, and TNF-*α* levels in the serum of patients with leptospirosis (*n*=57) and a healthy group (*n*=12) of individuals. The ELISAs were carried out following the manufacturer's instructions of each ELISA kit (Mabtech, Sweden). Mean absorbance values were calculated, and data were analyzed using Graphpad Prism version 6.05 (Graphpad Software Inc) to determine the level of cytokines.

### 2.5. Statistical Analysis

Mann–Whitney *U* test using 95% of confidence interval was used to determine the significant difference in cytokine levels between the leptospirosis patients and healthy control group. In order to compare the statistical significance between cytokine expression and renal failure or liver involvement, the Kruskal–Wallis test was used for post hoc analysis.

## 3. Results

### 3.1. Characteristics of the Study Sample

The study sample consisted of 57 patients with confirmed leptospirosis. The mean age of the patients was 43 ± 14 years. Occupation of the study samples was varied as farmers (26%), outdoor workers (24%), and indoor workers (23%). Exposure to contaminated environments was seen among 72%, while direct animal contact was seen among 42%. The mean duration of hospitalization ranged from 2 to 13 days. Among 57 patients, 21 had mild disease while 36 showed severe disease. Following complications were observed in the patient population which consisted of 7 patients with liver insufficiency, 14 patients with acute renal failure, 2 patients with disseminated intravascular coagulation (DIC), 4 patients with myocarditis, 2 patients with pulmonary oedema, and 3 patients with hypotension. Eight patients showed more than one complication.

### 3.2. Cytokine Concentrations

Among the five cytokines tested, IL-21 (*p*=0.002), IL-23 (*p*=0.002), and TNF-*α* (*p*=0.003) were significantly elevated among leptospirosis patients compared to the healthy controls (Table 1). The mean concentrations of IL-21, IL-23, and TNF-*α* among the leptospirosis patients were 14.69 pg/ml ± 13.1, 54.06 pg/ml ± 28.1, and 700 pg/ml ± 619, respectively. In the healthy control group, the mean concentration for each cytokine was 7.15 pg/ml ± 4.0, 21.75 pg/ml ± 17.7, and 62.95 pg/ml ± 37.2, respectively. Furthermore, there was a thread of increase in IL-10 (30.68 pg/ml ± 33.2) and IL-17A (11.66 pg/ml ± 5.4) in the leptospirosis group compared to the healthy group.

### 3.3. Association with Disease Severity

Table 2 describes the cytokine levels among leptospirosis patients with mild disease (without complications) and patients with renal failure, while Table 3 describes the cytokine levels among patients with mild disease and patients with liver insufficiency. The mean duration of days after the onset of illness in the mild group (without complications) was 4 days, while in the groups with renal failure or liver insufficiency was 9 days, respectively.

Patients who developed renal failure had significantly elevated levels of IL-21 (*p*=0.006) and IL-23 (*p*=0.002), while those having liver involvement had significantly elevated levels of IL-21 (*p*=0.001), IL-23 (*p*=0.001), and TNF-*α* (*p*=0.008) compared to the healthy controls (Tables 2 and 3). When the cytokine levels of patients with mild disease and renal failure were compared, IL-21 (*p*=0.001) and IL-23 (*p*=0.001) were significantly elevated. Significant elevation of cytokines IL-23 (*p*=0.003) and TNF-*α* (*p*=0.0009) was observed in patients with mild disease (no complications) and liver insufficiency.

## 4. Discussion

The host immune response is a major determinant of the outcome of leptospirosis. *Leptospira* in blood acts as extracellular bacteria and can stimulate various pathways of immune activation. T-helper 17 cells are known to play an important protective role in the immune response to extracellular bacterial and fungal infections. Activated Th17 cells produce proinflammatory cytokines IL-17 and TNF-*α* [7]. Two important cytokines involved in driving Th17 cell differentiation, regulation, and maintenance are IL-21 and IL-23 [8].

According to the present study, IL-21, IL-23, and TNF-*α* levels were significantly elevated among leptospirosis patients compared to the healthy controls. Interestingly, there was a significant elevation in IL-21 and IL-23 among leptospirosis patients with renal failure. Moreover, IL-21, IL-23, and TNF-*α* levels were significantly increased among leptospirosis patients with liver insufficiency.

In the present study, IL-17A levels were elevated in the leptospirosis group compared to the healthy controls, although no significant difference was seen between the two groups. It was of interest to note that when considering the patients with renal failure and liver failure, the IL-17 and IL-10 cytokine levels were lower compared to the healthy controls. The lower expression of IL-17 among these patients with complications may be due to the patients being in the recovering phase of the illness (more than 9 days in this study) where the inflammation is decreasing. This finding is supported by Papa et al. [2] who compared the cytokine expression levels in patients with the duration of illness and observed the declined expression of IL-10 and IL-17 with the number of days after the onset of illness.

Although IL-17A is mainly secreted by Th17 cells, innate immune cells like neutrophils and natural killer cells [9] also may produce IL-17. However, though not conclusive, the finding that IL-21 and IL-23, which are involved in the Th17 pathway, were significantly elevated together with high levels of IL-17 in this study suggests the possible involvement of TH17 pathway in the immune response in leptospirosis.

IL-17A induces the production of defensins, cytokines, and chemokines and can promote granulopoiesis and neutrophil recruitment [10]. Therefore, IL-17A induces proinflammatory cytokine and chemokine expression, resulting in inflammation and neutrophil infiltration for the elimination of the infecting pathogen [11]. While several studies have reported the presence of IL-17A in intracellular bacterial infections, only few studies have investigated the role of IL-17 in leptospirosis. Reis et al. [3] reported a significant difference in serum IL-17A expression between leptospirosis patients having mild symptoms who do not require hospitalization and patients having severe leptospirosis [3]. However, in the study by Papa et al., no significant difference in IL-17 levels was observed between the controls and patients presenting with mild or severe leptospirosis [2]. The results of the current study suggest a role of Th17 in the disease pathogenesis as described by Reis et al. [3] and Wang et al. [1].

IL-23 is important in promoting Th17 cell differentiation and is mainly produced by macrophages and dendritic cells in response to pathogens [12]. IL-23 is thought to be more critical for in vivo maintenance of Th17 cells [10]. IL-21 is capable of acting on Th17 cells in an autocrine manner in response to antigen stimulation. Thus, IL-21 serves as a factor that is produced by Th17 cells to promote or sustain their differentiation [13]. The significant elevation of IL-23, IL-21 and elevation of IL-17 observed in this study suggest a possible role of Th17 cells in the inflammatory response among patients with leptospirosis.

Another important proinflammatory cytokine elevated in leptospirosis is TNF-*α* which is generated predominantly from activated macrophages and also from nonimmune cells. Virulent *Leptospira* is a potent inducer of TNF-*α* via toll-like receptor dependent mechanism [14]. Neutrophil extravasation into tissues is increased in the presence of TNF-*α*. In combination with IL-1, TNF-*α* activates macrophages to secrete other proinflammatory cytokines and generates reactive oxygen and nitrogen species promoting the inflammation and tissue damage. Fatal leptospirosis has been associated with both high [15]and low levels of TNF-*α* [2] in different studies.

IL-10 is an anti-inflammatory cytokine mainly generated by regulatory T cells and to a lesser extent by the T-helper cell type 2 (Th2), macrophages, monocytes, natural killer cells, and B cells [16]. However, it is suggested that the elevation of IL-10 levels early in the disease is insufficient to prevent the tissue damage arising due to the inflammatory response. Patients with severe disease are reported to have higher IL-10 levels than patients with mild disease. IL-10 was reported to reach a peak within 1–5 days postinfection in a recent study among leptospirosis patients [2]. As the majority of patients with complications in the present study were more than 10 days after the onset of illness, the lower IL-10 cytokine concentrations may be due to the recovery phase.

Th17 cells have been shown to promote renal inflammation resulting in upregulation of chemokines and neutrophil infiltration leading to tissue injury. In mouse models in which nephrotoxic nephritis was induced by sheep serum, renal injury was associated with the presence of Th17 cells [17]. Furthermore, renal injury was minimized in IL-17 knock out mice injected with sheep serum [18]. However, the evidence for the role of Th17 cells in renal and liver involvement in leptospirosis is sparse and remains an enigma.

## 5. Conclusion

The current study demonstrates a pattern of five pro- and anti-inflammatory cytokines in human leptospirosis. It can serve as a basis for further well-designed studies with serial samples. However, elevation of IL-17 and significant elevation of IL-21, IL-23, and TNF-*α* in the present study suggest the involvement of Th17 cells in the immune response to *Leptospira*.

## Figures and Tables

**Table 1:** Comparison of serum cytokine levels in the leptospirosis patients and healthy control group.

Type of cytokine	Healthy group (pg/ml)		Leptospirosis group (pg/ml)		*p* value
	Range	Mean	Range	Mean	
IL-10	4–36.93	18.61 ± 10.5	4–166.5	30.68 ± 33.2	0.327
IL-17A	7–9.27	8.13 ± 0.6	0.003–16.93	11.66 ± 5.4	0.157
IL-21	1–14.11	7.15 ± 4.0	1–65.76	14.69 ± 13.1	0.002^*∗*^
IL-23	2–54.92	21.75 ± 17.7	5.94–132.95	54.06 ± 28.1	0.002^*∗*^
TNF-*α*	30.91–467.14	62.95 ± 37.2	30.91–2274.79	700 ± 619	0.039^*∗*^

^*∗*^ *p* < 0.05.

**Table 2:** Comparison of mean serum cytokine levels in the healthy control group, leptospirosis patients without renal failure, and leptospirosis patients with renal failure.

Cytokine	Control (pg/ml)	Leptospirosis patients without renal failure (pg/ml)	Leptospirosis patients with renal failure (pg/ml)	*p* value healthy-mild	*p* value healthy-with renal failure	*p* value without renal failure-with renal failure
IL-10	18.61 ± 10.5	17 ± 4.3	11.43 ± 11.8	NS	NS	NS
IL-17A	8.13 ± 0.6	13.3 ± 6.2	4.43 ± 4.6	NS	NS	NS
IL-21	7.15 ± 4.0	9.21 ± 5.0	15.74 ± 9.2	NS	0.006^*∗*^	0.001^*∗*^
IL-23	21.75 ± 17.7	32.2 ± 16.2	56.20 ± 17.1	NS	0.002^*∗*^	0.001^*∗*^
TNF-*α*	62.95 ± 37.2	300.52 ± 200.2	422.48 ± 373.1	0.0002^*∗*^	NS	NS

^*∗*^ *p* < 0.016, NS; not significant.

**Table 3:** Comparison of mean serum cytokine levels in leptospirosis patients with liver insufficiency, mild disease without complication, and healthy control group.

Type of cytokine	Mean value of healthy group (pg/ml)	Mean value of patients (pg/ml)	Mean value of leptospirosis patients with liver involvement (pg/ml)	*p* value healthy-without liver insufficiency	*p* value healthy-with liver insufficiency	*p* value without liver insufficiency-with liver insufficiency
IL-10	18.61 ± 10.5	20 ± 4.2	7.91 ± 9.4	NS	NS	NS
IL-17A	8.13 ± 0.6	4.3 ± 4.3	5.78 ± 5.5	NS	NS	NS
IL-21	7.15 ± 4.0	13.53 ± 6.1	16.74 ± 8.3	0.001^*∗*^	0.001^*∗*^	NS
IL-23	21.75 ± 17.7	45.87 ± 15.2	64.78 ± 15.8	0.0001^*∗*^	0.001^*∗*^	0.003^*∗*^
TNF-*α*	62.95 ± 37.2	450 ± 300.5	702.84 ± 532.3	0.0001^*∗*^	0.008^*∗*^	0.0009^*∗*^

^*∗*^ *p* < 0.016; NS, not significant.

## Data Availability

The data used to support the findings of this study are available from the corresponding author upon request.
